# New Medical Journals

**Published:** 1866

**Authors:** 


New Medical Journals.—We welcome to our exchange list the following medical journals, all of which are ably conducted, instructive and valuable medical periodicals: Atlanta Medical and

Surgical Journal, Detroit Review, St. Louis Medical Reporter, Galveston Medical Journal.

We have received, also, prospectus of Southern Journal of the Medical Sciences, to be published in New Orleans, to contain 200 pages per number, and to be issued quarterly.

We are gratified at these evidences of returning prosperity in the profession at the South, and hope that from restored peace and union, may spring all the blessings which flow from wise and beneficent government, prosperous agriculture, extensive commerce, and faithfully cultivated science.

